# APOE4 alters the regional levels of numerous neuroimaging biomarkers across the Alzheimer's disease continuum

**DOI:** 10.1002/alz70856_103473

**Published:** 2025-12-25

**Authors:** Jason A Mares, Jia Guo, Tal Nuriel

**Affiliations:** ^1^ Columbia University, New York, NY, USA; ^2^ Zuckerman Institute, Columbia University, New York, NY, USA; ^3^ Columbia University Irving Medical Center, New York, NY, USA; ^4^ Taub Institute for Research on Alzheimer's Disease and the Aging Brain, New York, NY, USA; ^5^ Department of Cell Biology and Pathology, New York, NY, USA

## Abstract

**Background:**

Carriers of the apolipoprotein E ε4 (*APOE4*) allele are at an increased risk of developing Alzheimer's disease (AD) and display an earlier age of onset compared to non‐carriers who develop the disease. While researchers have extensively characterized the impact of *APOE4* on the susceptibility of AD, little is known about the differences in disease etiology and presentation in *APOE4* carriers vs. non‐carriers.

**Method:**

In order to investigate these differences, we performed a broad analysis of neuroimaging biomarkers in *APOE4* carriers vs. non‐carriers present in the ADNI cohort. We also have analyzed this data using a novel deep learning method called DeepContrast, which uses artificial intelligence to identify functional brain activity signatures in structural MRI scans.

**Result:**

We observed differences between *APOE4* carriers vs. non‐carriers in several neuroimaging readouts, including Ab‐PET, Tau‐PET, FDG‐PET, structural MRI, and FLAIR MRI. These differences were variable based on sex and age. Interestingly, when we compared cognitively unimpaired ADNI participants who converted to MCI or AD to those who did not, we observed *APOE4*‐dependent differences that may point to specific pathologies that mediate MCI/AD conversion in *APOE4* carriers vs. non‐carriers. In addition, we also have exciting new data showing distinct differences in *APOE4* carriers vs. non‐carriers from ADNI using DeepContrast.

**Conclusion:**

These observations of pathological heterogeneity between *APOE4* carriers vs. non‐carriers reveal significant differences in disease pathogenesis between these two groups, which may have important implications with regard to the diagnosis and treatment of AD in different at‐risk populations.

